# The role of Wnt signalling in development of coronary artery disease and its risk factors

**DOI:** 10.1098/rsob.200128

**Published:** 2020-10-21

**Authors:** Ya Liu, Arpita Neogi, Arya Mani

**Affiliations:** 1 Department of Cardiology, West China Hospital, Sichuan University, Chengdu, People's Republic of China; 2 Yale Cardiovascular Genetics Program, Yale University, New Haven, CT, USA; 3 Yale Cardiovascular Research Center, Department of Medicine, Yale University, New Haven, CT, USA; 4 Department of Genetics, Yale University School of Medicine, Yale University, New Haven, CT, USA

**Keywords:** Wnt signalling, atherosclerosis, coronary artery disease, pathology

## Abstract

The Wnt signalling pathways are composed of a highly conserved cascade of events that govern cell differentiation, apoptosis and cell orientation. Three major and distinct Wnt signalling pathways have been characterized: the canonical Wnt pathway (or Wnt/β-catenin pathway), the non-canonical planar cell polarity pathway and the non-canonical Wnt/Ca^2+^ pathway. Altered Wnt signalling pathway has been associated with diverse diseases such as disorders of bone density, different malignancies, cardiac malformations and heart failure. Coronary artery disease is the most common type of heart disease in the United States. Atherosclerosis is a multi-step pathological process, which starts with lipid deposition and endothelial cell dysfunction, triggering inflammatory reactions, followed by recruitment and aggregation of monocytes. Subsequently, monocytes differentiate into tissue-resident macrophages and transform into foam cells by the uptake of modified low-density lipoprotein. Meanwhile, further accumulations of lipids, infiltration and proliferation of vascular smooth muscle cells, and deposition of the extracellular matrix occur under the intima. An atheromatous plaque or hyperplasia of the intima and media is eventually formed, resulting in luminal narrowing and reduced blood flow to the myocardium, leading to chest pain, angina and even myocardial infarction. The Wnt pathway participates in all different stages of this process, from endothelial dysfunction to lipid deposit, and from initial inflammation to plaque formation. Here, we focus on the role of Wnt cascade in pathophysiological mechanisms that take part in coronary artery disease from both clinical and experimental perspectives.

## 1. Introduction

**Figure 1.**
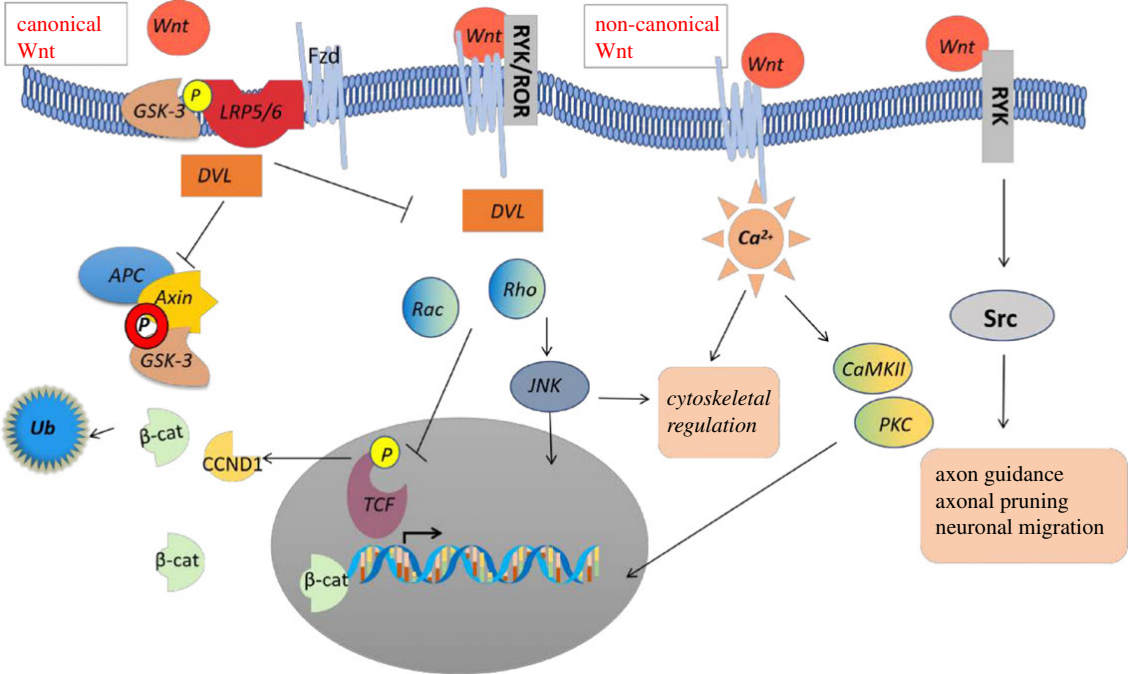
A schematic of canonical Wnt versus non-canonical signalling.

The Wnt signalling cascade is a highly conserved signalling cascade in stem cells and progenitor cells, regulating cell differentiation, apoptosis and cell orientation during embryonic development. It has a bi-directional regulatory effect at crucial aspects of cardiovascular development, including the formation of the heart tube, cardiac looping, chamber formation, as well as septation and maturation [1]. Wnt signalling is a developmental programme that is highly active during embryogenesis but also remains active in a subset of proliferating adult cells [2]. The malfunctioning of the Wnt signalling pathway during adult life has been associated with cardiac diseases as divergent as myocardial infarct healing, hypertrophy, heart failure, arrhythmias and atherosclerosis [3,4]. Three distinct pathways have been characterized: the canonical Wnt pathway (or Wnt/β-catenin pathway), the non-canonical planar cell polarity (PCP) pathway and the non-canonical Wnt/Ca^2+^ pathway. By binding of a Wnt protein ligand to a member of Frizzled (Fzd) family of receptors, these pathways play a part in transmitting the biological signal into the cell. In the canonical pathway, Wnt ligands bind to the Fzd receptor and low-density lipoprotein (LDL) receptor-related protein (LRP) complex 5/6 (LRP5/6) co-receptors, resulting in the accumulation of β-catenin in the nucleus, thus regulating downstream transcription. The non-canonical cell polarity pathway is thought to regulate the cytoskeleton by activating the derailed receptor tyrosine kinase (RYK)/receptor tyrosine kinase-like orphan receptor (ROR) co-receptors and activating the Rho and Ras-related C3 botulinum toxin substrate. The stimulation of calcium signalling non-canonical pathway activates phospholipase C, thus leading to calcium release [5] (figure 1).

**Table 1.** Anti- and proatherogenic functions of the Wnt signalling molecules.

Wnt signalling members	cell type/model system	phenotype	references
TCF7L2	high cardiovascular risk population, patients with T2DM, SMCs	rs7903146 associated with a higher prevalence and severity of CAD, lipid levels and angiographically diagnosed CAD; promote differentiation and inhibit proliferation of SMCs	Bielinski *et al*. [37]; Sousa *et al*. [38]; Muendlein *et al*. [41]; Srivastava *et al*. [74]
Dkk-1	T2DM/ ischaemic patients; patients with acute coronary syndromes/ischaemic stroke; HUVECs, ApoE^−/−^ mice; aortic ECs	no correlation with stroke severity and cardiovascular events; inversely associated with CAD or atherosclerosis; platelet-mediated ECs activation and inflammatory cytokines release; endothelial–mesenchymal transition	Gaudio *et al*. [42]; Seifert-Held *et al*. [43]; Ueland *et al*. [64]; Zhu *et al*. [46]; Ueland *et al*. [45]; Souilhol *et al*. [66]; Cheng *et al*. [67]
LRP6	early onset familial CAD and MetS, LRP6 knock down cells, LRP6 R611C mice, HEK293T^ΔLRP6^, LDLR^−/−^ mice, SMCs	LRP6 mutations impaired LDL clearance; increased hepatic de novo lipogenesis; ANGPTL4 inhibits Wnt signalling by decreasing LRP6 levels; LRP6-KO in SMCs promote aortic calcification	Mani *et al*. [12]; Ye *et al*. [54]; Go *et al.* [55]; Kirsch *et al*. [56]; Cheng *et al*. [80]
Wnt5a	atherosclerotic patients and murine, HUVECs, microphages	high circulating levels, advanced arterial lesions and microphage-rich region; positively correlated to triglyceride levels, vascular insulin resistance, and endothelial dysfunction; stimulate microphage pro-inflammatory cytokines and chemokines release	Christman *et al*. [59]; Bretón-Romero *et al*. [57]; Relling *et al*. [61]; Bhatt *et al*. [82]
Dkk-3	Dkk-3^−/−^/ApoE^−/−^ mice, HUVECs, SMCs	aortic endothelial damage; endothelium, SMC loss in the Dkk-3^−/−^/ApoE^−/−^ mice; Dkk-3 induced endothelial ECs migration; post-injury repair and angiogenic program; SMC and EMC deposition	Yu *et al*. [31]; Busceti *et al*. [86]; Karamariti [32]
LRP5	microphages, LRP5^−/−^ mice	high expression in advanced plaques; larger aortic atherosclerotic lesions and higher macrophage infiltration; upregulated cytokines and pro-inflammatory genes in HC LRP5^−/−^ mice	Ohta *et al*. [83]; Borrell-Pagès *et al.* [87,88]

Coronary artery disease (CAD) is the leading cause of death worldwide, which is usually caused by the rupture of an atherosclerosis plaque within the coronary arteries or the erosion of the endothelium and superimposed thrombosis [6]. It is a pathological condition which is primarily caused by atherosclerosis, wherein the formation of plaque within the artery walls causes narrowing of the arteries and slows down blood flow over time. This process starts with lipid deposition and endothelial cell (EC) dysfunction that subsequently induces the secretion of inflammatory cytokines, and the recruitment, adherence and aggregation of monocytes into the subendothelial space of the arterial wall. These monocytes further differentiate into macrophages and then into foam cells by the uptake of modified LDL. Meanwhile, further accumulation of lipids, infiltration and proliferation of vascular smooth muscle cells (VSMCs), as well as deposition of the extracellular matrix (ECM) exacerbate inflammation and adaptive immunity reaction under the thickened intima. Plaque erosion is characterized by VSMC proliferation and endothelial defects in young people, both male and female, which can later result in endothelial layer erosion and superimposed thrombosis [7]. An atheromatous plaque is eventually formed inside the vessel lumen, inhibiting blood supply; this can lead to angina and myocardial infarction (MI) due to coronary artery thrombosis. During the final stage, reduced ECM deposition, SMC apoptosis and non-programmed cell death make the atheromatous plaques less stable, thereby increasing the risk of rupture and thrombosis [8,9]. In addition, coronary artery thrombosis has been shown to occur in young patients without advanced atherosclerosis. The examination of coronary artery autopsy specimens in these subjects has shown thrombosis which occurs on a substrate of endothelial erosion and VSMC proliferation [10]. The Wnt/β-catenin pathway participates in different stages of this process. Moreover, the canonical Wnt/β-catenin pathway plays a key role in both cell fate decision and initial inflammation of atherosclerosis, as well as the uptake and internalization of LDL and the formation of additional foam cells [11].

Metabolic syndrome (MetS), a condition characterized by hyperlipidaemia, hyperglycaemia, hypertension and obesity, is associated with a high risk for cardiovascular disease. Previously, our group performed genetic linkage analysis in a large outlier family with a high prevalence of early onset CAD and MetS, leading to the discovery of a novel non-conservative mutation in a gene that encodes Wnt-coreceptor LRP6. The mutation, which leads to p.R611C substitution, completely segregated with CAD and metabolic traits of insulin resistance or diabetes, hypertension, hyperlipidemia, truncal obesity and osteoporosis [12]. This was the first report implicating altered Wnt signalling in atherosclerosis and MetS. Since this discovery, independent studies have shown that the Wnt components and its antagonist, serum Dickkopf-related proteins (Dkk), play an important role in the development of coronary atherosclerosis. Here, we review the involvement of Wnt signalling in CAD pathology from both clinical and experimental perspectives.

## 2. Wnt molecules, the signalling cascades, natural activators and inhibitors

Wnt genes were first identified in mutant wingless *Drosophila melanogaster* in 1978 and were then named Wingless genes [13]. Int1 was initially identified as an activated proto-oncogene in mouse breast cancer. Subsequent sequencing evidence confirmed Int1 and Wingless were in fact homologous and those two terms were eventually combined in 1982 to form a fusion name: ‘Wnt’ [14]. The Wnt cascade is highly conserved and is active in many tissue types throughout the life cycle. Additionally, post-natal Wnt signalling regulates numerous biological processes in proliferative organs such as the gut [15], breast and skin, and in pathological processes involved in bone disorders, metabolic disorders, inflammatory disorders and cancer [16].

The Wnt gene family consist of 19 structurally related genes which encode cysteine-rich secreted glycoproteins, acting as extracellular signalling factors in mammals. Wnt protein family can be further divided into either canonical or non-canonical categories, yet there is significant overlap between the two: canonical Wnt includes Wnt1, Wnt2, Wnt3, Wnt8 and Wnt10, and non-canonical Wnt includes Wnt4, Wnt5, Wnt6, Wnt7 and Wnt11. However, canonical and non-canonical Wnts are capable of activating unrelated coreceptors through a shared mechanism [17]. Growing evidence indicates overlapping function between those Wnt proteins; for example, wnt5a [18] and Wnt 11 [19] both act ‘canonically' and ‘non-canonically’. The canonical Wnt signalling is activated after Wnt binds to G-coupled protein receptors of Fzd family and their coreceptors LRP5/6 to prevent β-catenin destruction and its transportation to the nucleus for co-transcription of Wnt-regulated genes (figure 1). Ten Fzd receptors have been identified to this date. The Fzd family members are further divided into five subfamilies, namely Fzd1/2/7, Fzd5/8, Fzd9/10, Fzd4 and Fzd3/6, according to structural similarity. β-catenin destruction complex is composed of Axin, adenomatous polyposis coli (APC) and casein kinase1 *α*(CK1*α*), together with glycogen synthase kinase 3 (GSK-3) which phosphorylate β-catenin, tagging it for ubiquitination. Binding of Wnt to Fzd and LRP5/6 in the canonical Wnt pathway causes the disintegration of a cytoplasmic β-catenin destruction complex, resulting in the accumulation of active (non-phosphorylated) β-catenin, which then translocates from the cytoplasm to the nucleus, binding to the transcription factor T-cell factor (TCF) and/or lymphoid enhancer factor (LEF) to affect target gene transcription. In specific contexts, Wnt/β-catenin signalling triggers the simultaneous activation of Wnt/JNK. In mammals, over 50 genes have been identified that are modulated by this pathway, including genes involved in the Wnt transduction pathway itself, providing a feedback loop [20]. Wnt proteins also activate β-catenin-independent pathways that are collectively known as non-canonical Wnt signalling, including Wnt/Ca^2+^ pathway and the PCP pathway. Ca^2+^ is one of the most abundantly distributed second messengers, triggering physiological activities like muscle contraction, endocrine response and cell fate determination. Activation of the calcium pathway facilitates Ca^2+^ release from the endoplasmic reticulum in a G-protein-dependent manner [21]. Calcium calmodulin-dependent kinase II (CaMKII) and protein kinase C (PKC) are downstream effectors of Wnt/Ca^2+^ signalling activation [22]. The PCP pathway is activated through the binding of Wnt ligands to the Fzd receptors such as RYK and ROR2, and activates the Rho and Rac GTPases, leading to cytoskeletal reorganization and coordinating morphogenetic cell behaviours during gastrulation and neurulation. In addition, RYK signalling controls axon guidance, axonal pruning and neuronal migration through an Src family-dependent pathway [23].

There are several natural inhibitors for Wnt signalling pathways, such as secreted Fzd-related proteins (sFRPs), Wnt inhibitory factors (WIFs) and/or Dkk family. But beyond that, sFRPs and WIFs may also affect extracellular Wnt stabilization through Wnt-independent ways, such as the regulation of extracellular proteinases. Studies have found that sFRPs could interfere with bone morphogenetic protein signalling by acting as proteinase inhibitors [24]. The vertebrate Dkk family consists of four members, named Dkk–1–4, sized between 255 and 350 amino acids. Dkk-1, 2 and 4 share two conserved cysteine-rich domains. Dkk-1 selectively blocks Wnt/β-catenin pathway by forming a ternary complex with LRP5/6. Conversely, Dkk-1 itself is a target of canonical Wnt signalling pathways, thereby establishing a negative feedback loop [25]. However, it has been suggested that Dkk-1 and Dkk-2 might have β-catenin-independent functions. Overexpression of Dkk-1 induced apoptosis and growth suppression in β-catenin-deficient mesothelioma cell lines H28 and MS-1. Furthermore, the inhibition of JNK rescued the apoptosis induced by Dkk-1 overexpression in these cells [26]. Similarly, in HeLa cells, ectopic expression of Dkk-1 had no effect on either cellular β-catenin localization or TCF-reporter activity [27]. These data suggest that Dkk-1 might be capable of exerting its suppressive effects by antagonizing Wnt signalling through β-catenin-independent pathways. Unlike Dkk-1, Dkk-2 is able to act either as blocker or activator of the Wnt pathways, depending on the cellular context [28]. Dkk-2 strongly synergizes with Wnt receptors of the Fzd family to induce Wnt signalling when overexpressed in *Xenopus* embryos [29]. Dkk-2 stimulated LRP6 and Wnt/Fzd signalling activation in human 293T cells. However, the co-transfection of Kremen2 (Krm2) blocked the stimulating effect of Dkk-2 and enhanced inhibition of the Wnt pathway [30]. Some evidence has suggested that Dkk-3 can display an atheroprotective role either by the activation of the transforming growth factor-β and Wnt signalling pathways or β-catenin-independent pathways [31,32]. Conversely, Cheng *et al*. [33] demonstrated Dkk-3 ablation attenuated the development of atherosclerosis in ApoE-deficient mice fed with high-fat diet through the activation of β-catenin signalling. At the present time, there is insufficient data on Dkk-4 protein [34]; even though its role has been recently implicated in colorectal cancer, hepatocellular carcinoma and pancreatic cancer, there is limited evidence on atherosclerosis.

## 3. Clinical evidence for the role of Wnt signalling in coronary artery disease

Common genetic variants of TCF7L2 have been reproducibly associated with the risk for type 2 diabetes (T2DM) in multiple genome-wide association studies [35,36]. T2DM is a major risk factor for stroke and CAD (table 1). However, the information on whether and how TCF7L2 polymorphisms alter lipids metabolism and cause cardiovascular disease is incomplete and remains controversial. In the atherosclerosis risk in communities (ARIC) study, 13 369 black and white subjects who were free of cardiovascular disease (CVD) at baseline were genotyped for the SNPs rs7903146, rs12255372, rs7901695, rs11196205 and rs7895340. In these subjects, TCF7L2 was not significantly associated with the development of CAD, ischaemic stroke, CVD or all-cause mortality [37]. On the contrary, a randomized controlled trial which evaluated the association between TCF7L2 polymorphism and CAD in a high cardiovascular risk population found that rs7903146 (allele T) was associated with a higher prevalence and severity of CAD and cardiovascular events in non-diabetic individuals [38]. Kucharska-Newton *et al.* performed further analysis of the ARIC study and reached similar conclusions that body mass modified the association of the TCF7L2 rs7903146 T allele with CAD risk [39]. Additionally, a study suggested the TCF7L2 rs7903146 polymorphism was associated with T2DM for TT instead of CC individuals: Mediterranean diet (MedDiet) interacted with rs7903146 on fasting glucose as well as blood lipid levels [40]. A recessive interaction model showed the statistical significance of gene–diet interactions in determining plasma total cholesterol, LDL-C and triglyceride levels. Higher plasma concentrations of total cholesterol, triglycerides and LDL-C were observed in TT individuals when adherence to the MedDiet was low. However, when adherence to the MedDiet was high, these effects no longer existed, and there were no differences in circulating lipid parameters between genotypes [40]. In a cross-sectional study [41] including 1650 consecutive patients undergoing coronary angiography, TCF7L2 variants rs7903146, rs12255372 and rs11196205 were significantly associated with angiographically diagnosed CAD, specifically in patients with T2DM. Thus, TCF7L2 was projected as a potential genetic link between diabetes and atherosclerosis-related disease.

Clinical studies aiming at the impact of Dkk-1 on atherosclerosis have also generated controversial results, showing either direct or inverse correlations (table 1). Gaudio *et al*. evaluated the association of circulating sclerostin and Dkk-1 with carotid intima-media thickness (CITM) in T2DM patients. Serum concentrations of sclerostin and Dkk-1 were significantly higher in the T2DM group compared with controls. However, these associations no longer existed for Dkk-1 after adjustment for potential confounders [42]. Further negative clinical evidence came from Seifert-Held *et al.* who found Dkk-1 levels had no correlation with stroke severity, and circular concentrations of Dkk-1 did not differ significantly between subtypes of ischaemic stroke [43]. Consistent with that study, Ress *et al*. found no systemic Dkk-1 level changes in patients with cardiovascular events [44]. On the contrary, more recent studies have suggested that Dkk-1 is inversely associated with CAD or atherosclerosis. Studies indicated that high serum Dkk-1 level is associated with ischaemic stroke and cardiovascular death consistently [45]. A large clinical study [46] which enrolled 3178 patients with ischaemic stroke from the China Antihypertensive Trial in Acute Ischemic Stroke (CATIS) reported an association between high baseline serum Dkk-1 levels and poor prognosis one year after ischaemic stroke, suggesting that circular Dkk-1 might be a potential prognostic indicator for ischaemic stroke.

## 4. Wnt signal in lipid metabolism

Accumulating evidence has described a critical role for Wnt in the process of lipid storage and homeostasis, including adipogenesis, intracellular cholesterol trafficking and cholesterol egress [47]. As explained, genetic investigations of families or populations with extreme forms of MetS and/or early onset CAD have led to the discovery of both rare and common mutations in *LRP6*. These mutations were associated with the risk of elevated LDL-cholesterol (LDL-C) [48]. A single missense mutation in Wnt co-receptor LRP6, p.R611C, was first reported as the underlying cause of autosomal dominant early onset CAD and multiple metabolic risk factors including hypertension, hyperlipidaemia and diabetes in a very large Iranian family [12]. Three other rare mutations in LRP6 (p.R360H, p.R473Q and p.N433S) were later found in the white American population with early onset familial CAD and MetS [49]. A new LRP6 p.Y418H mutation was found to contribute to normolipidaemic familial CAD via impairing endothelial cell functions [50]. Common variants of LRP6 gene such as rs10845493 have been associated with elevated LDL-C, whereas *LRP6* variant rs2302685 has been linked to increased risk of MI in the Chinese population [51]. This association was even stronger among younger populations. Likewise, LRP6 p.I1062V has been associated with the presence of carotid artery atherosclerosis in hypertensive patients [52].

**Figure 2.**
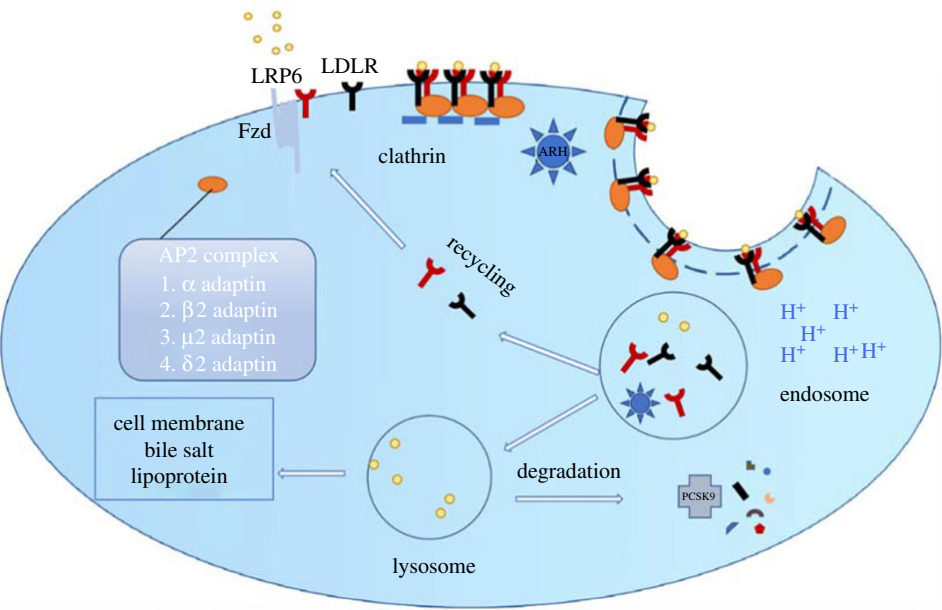
LDL internalization by clathrin-dependent endocytosis.

Among all *LRP6* variants, the role of LRP6 p.R611C in lipid metabolism is most thoroughly studied. LDL uptake was significantly lower in the lymphoblastoid cells of the LRP6 p.R611C carriers compared with unaffected family members. Reduced LRP6 expression and LDL uptake were also found in splenic B cells of LRP6^+/−^ mice compared with their wild-type (WT) littermates [53]. *In vitro* evidence showed that LRP6 knockdown in LDLR-deficient CHO cells caused a modest reduction of LDL binding and uptake, while its knockdown in regular CHO cells resulted in a much greater decline in LDL levels, suggesting an interaction between LRP6 and LDLR. Loss of LRP6 resulted in severely diminished LDLR internalization which could be rescued after reintroduction of LRP6. It was shown that LRP6 (WT) forms a complex with LDLR and autosomal recessive hyperlipidaemia protein (ARH), which upon stimulation with LDL undergoes a clathrin-mediated internalization process (figure 2). LDLR and LRP6 internalization as well as LDL uptake were also impaired in LRP6 p.R611C CHO cells. These results suggested that LRP6 is involved in LDL clearance [54]. However, impaired LDL clearance could not alone explain the severity of plasma cholesterol elevation in LRP6 p.R611C. Go *et al*. generated a transgenic mouse model of LRP6^R611C^ to examine hepatic lipid synthesis [55]. LRP6 ^R611C^ mice exhibited elevated plasma LDL, TG levels and fatty liver. Further investigation showed that LRP6^R611C^ mutation triggers hepatic de novo lipogenesis, cholesterol biosynthesis and ApoB secretion by activation of IGF1, AKT and mTOR1/2 pathways. *In vitro* treatment of hepatocytes with either the IGF1 receptor antagonist, rapamycin or rmWnt3a could normalize disease pathways in LRP6^R611C^ mice. The administration of rmWnt3a to mutant mice could further normalize cholesterol biosynthesis and restore normal plasma TG and LDL levels [55]. Interestingly, angiopoietin-like 4 (ANGPTL4) [56] has been identified as a Wnt signalling antagonist that forms a ternary complex with LRP6. ANGPTL4 regulates lipid metabolism via attenuating the clearance of circulating triglycerides by the inhibition of lipoprotein lipase. This protein forms a complex with LRP6, triggering its internalization via clathrin-mediated endocytosis and its degradation in lysosomes, leading to attenuation of Wnt/β-catenin signalling.

## 5. Wnt and endothelial dysfunction

Endothelial dysfunction is regarded as an initial step in the pathogenesis of the vascular disease. The contribution of Wnt5a to endothelial dysfunction and immunity reaction has been highlighted recently [57,58]. Wnt5a is a secreted glycoprotein member of the Wnt family. Circulating Wnt5a was found to be higher in atherosclerotic patients than in healthy controls. Wnt5a transcripts and protein were also elevated in advanced arterial lesions [54]. Immunohistochemical analysis indicated a higher expression of Wnt5a in the macrophage-rich regions in both human and murine atherosclerotic lesions [59]. Wnt5a acted as a vital inflammatory mediator in the process of atherosclerosis by inducing rapid inflammatory gene expression, including GM-CSF, IL-1a, IL-3, IL-5, IL-6, IL-7, IL-8, CCL2, CCL8 and COX-2 in human aortic ECs. The induction of COX-2 by Wnt5a was endothelial specific and was not observed in non-endothelial cells. Interestingly, the subset of cytokines regulated by Wnt5a and TNF-alpha were only partially overlapping, suggesting that endothelial inflammation response was under the regulation of a dual system, namely non-canonical Wnt pathway and TNF-alpha-mediated signalling [60]. Recent data show Wnt5a signalling also positively correlates with triglyceride levels, vascular insulin resistance and endothelial dysfunction [61,62]. mRNA level of Wnt5a has been shown to be upregulated in ECs treated with a combination of inflammatory cytokines including TNF-a, IL-1 and IL-8. The role of Wnt5a in the regulation of human EC proliferation and migration was examined by using a goat polyclonal anti-Wnt5a neutralizing antibody. Wnt5a antibody inhibited HUVECs proliferation in a dose-dependent manner, which was reversed by VEGF treatment. Blocking Wnt5a/Ca^2+^ signalling by antibody also inhibited endothelial cell migration. The same suppression effect was observed by siRNA or a downstream inhibitor [63].

Ueland *et al.* reported that Dkk-1 could be operating within the atherosclerotic lesion as a novel mediator in platelet-mediated ECs activation and Dkk-1-driven inflammatory loop [64]. They reported systemic and lesion-specific elevation of Dkk-1 in ApoE^−/−^ mice, in human subjects with CAD and in patients with carotid plaque atherosclerosis. Immunostaining of thrombus material obtained from ruptured plaque further identified platelets as an essential source of Dkk-1. Dkk-1 could induce endothelial activation and inflammatory cytokine release via inhibition of Wnt signalling pathway and activation of NF-kB. Blocking Dkk-1 totally abolished mRNA and protein expression of MCP-1 and IL-8 in HUVECs after platelet stimulation [64]. In another study, knock-in of Dkk-1 gene in ApoE^−/−^ mice resulted in enlarged and destabilized atherosclerotic lesions, while silencing of it alleviated plaque formation and vulnerability. Dkk-1 expression was upregulated in response to oxidized low-density lipoprotein (ox-LDL) treatment in a time- and concentration-dependent manner in HUVECs [65]. Furthermore, Dkk-1 regulated the endothelial–mesenchymal transition in aortic ECs, a critical event that drives the initiation and progression of atherosclerosis [66,67]. Together, those data provide convincing evidence considering Dkk-1 as a Wnt/β-catenin inhibitor with a proatherogenic role.

In the prospective population-based Bruneck Study, circular Dkk-3 levels were inversely associated with carotid artery intima-media thickness, raising the hypothesis that Dkk-3 could confer protection against multiple pathological stages of atherogenesis by inhibiting the non-canonical Wnt. Dkk-3^−/−^/ApoE^−/−^ mice exhibited larger endothelial damage and apparent endothelium loss in the aorta. Transwell and scratch migration assays exhibited a significant induction of cultured human EC migration in response to Dkk-3 stimulation. The mechanism underlying this effect might be the activation of the non-canonical Wnt pathway; more specifically, the ROR2-DVL1-Rac1-JNK signalling pathways. This study suggested a protective role of Dkk-3 in the context of atherosclerosis progression by inducing endothelial migration and post-injury repair. Moreover, Dkk-3 deficiency also showed a delayed reendothelialization and aggravated neointima formation in a wire-injured mouse model [31]. Dkk-3 stimulation has been reported to trigger the angiogenic programme *in vitro* by augmenting intracellular and extracellular VEGF protein levels. Dkk-3 recruited Smad4 binding to gene promoter regions, resulting in a transcriptional activation of VEGF and induction of the angiogenic programme in HUVECs [68].

## 6. Wnt signalling alters proliferation, migration and apoptosis of smooth muscle cell

Normally, SMCs reside in the medial layer of arteries, showing a contractile phenotype with low proliferative rates. During atherosclerosis progression, ECs dysfunction and the associated inflammatory state trigger the de-differentiation and phenotypic switching of SMCs. Those SMCs exhibiting a synthetic phenotype subsequently migrate into the intima where they further proliferate and deposit ECM. Paradoxically, SMC proliferation is considered beneficial during later stages of fibrous cap formation, which renders resistance against plaque rupture. As the disease progresses, decreased ECM synthesis together with SMC apoptosis results in the reduction of fibrous cap tensile strength.

SMC proliferation triggered by platelet-derived growth factor (PDGF) in response to endothelial injury has been reported [69]. Inhibition of PDGF receptor-beta has been associated with increased aortic atherosclerotic lesion size and the number of intimal SMCs by 67% and 80%, respectively [70]. Activation of Wnt/β-catenin signalling altered the expression of pro-proliferative genes including cyclin D1 and p21 in both arterial and venous SMCs [71]. Wnt1 and Wnt3a were critical regulators of β-catenin signalling and cyclin D1 expression in arterial SMCs [72], while Wnt4 was capable of inducing of arterial SMC proliferation and contributed to pathological intimal thickening. *In vitro* studies indicated Dkk-3 was able to induce differentiation of vascular progenitors and fibroblasts into SMCs via activation of the transforming growth factor-β/activating transcription factor 6 and Wnt signalling pathways. Larger and more vulnerable atherosclerotic lesions with more macrophages, reduced number of SMCs and less EMC deposition were found in Dkk-3^−/−^/ApoE^−/−^ mice under a chow diet in comparison with Dkk-3^+/+^/ApoE^−/−^ mice. Most importantly, exogenous recombinant Dkk-3 was capable of improving the composition of carotid atherosclerotic plaques in Dkk-3^−/−^/ApoE^−/−^ mice fed a high-fat diet by reducing intraplaque haemorrhage and macrophage infiltration, while increasing SMCs and EMC deposition [32]. On the other hand, impaired Wnt signalling has also been shown to induce VSMC proliferation. LRP6^R611C^ mice exhibited severe vascular obstruction after carotid injury and dramatic obstructive CAD under high-fat diet. Loss of VSMC differentiation and aortic medial hyperplasia were induced by diminished TCF7L2 expression, enhanced non-canonical Wnt signalling and Sp1-dependent activation of PDGF signalling. Those Wnt/β-catenin signalling suppression effects could be firmly rescued by Wnt3a administration [73]. Injury-induced intimal hyperplasia could similarly be rescued by overexpression of TCF7L2 in LRP6 mutant mice. TCF7L2 promoted differentiation and inhibited proliferation of SMCs by stabilization of GATA-binding protein 6 and upregulation of SMC myosin heavy chain and cell cycle inhibitors [74]. These findings demonstrate a causal link between LRP6/TCF7L2 and VSMC plasticity, indicating the crucial role of intact Wnt signalling in arterial disease. Thus, Wnt signalling appears to be a delicately balanced pathway, and it both increased and reduced activation, triggering the vascular disease.

The role of Wnt proteins in SMC migration is not well understood. Williams *et al*. [75] demonstrated that Wnt2 regulates SMC migration and triggers intimal thickening *in vitro*. Indirect evidence suggested that non-canonical Wnt pathways may also be involved in SMC migration [76,77]. Recent findings suggested knockdown of peptidyl-prolyl cis/trans isomerase (Pin1) effectively resulted in cell cycle arrest, SMC apoptosis, thinning of the fibrous cap and increasing the incidence of plaque rupture. The β-catenin/cyclin D1/CDK4 cascade and mitochondrial pathway were shown to regulate SMC apoptosis. Moreover, transfection of a degradation-resistant β-catenin transgene into rat SMCs significantly inhibited apoptosis. Accumulation of β-catenin resulted in a higher expression of TCF. Dominant-negative TCF-4 transgene lacking the β-catenin binding domain could abolish β-catenin induced cell survival and cyclin D1 activation, and partially block G1-S phase transition at the same time [78]. Towler's group has shown that canonical Wnt signals mediate the vascular calcification of Msx2 transgenic mice in a paracrine fashion [79]. Several years later they demonstrated enhanced aortic osteochondrogenic programmes and increased circulating osteopontin in SMC(SM22)-specific LRP6-KO mice on LDLR^−/−^ background compared with LRP6(fl/fl);LDLR(-/-) controls. Loss of LRP6 in SMC promoted aortic calcification through activation of non-canonical Wnt signals [80]. Mass spectrometry identified LRP6 binding to protein arginine methyltransferase (PRMT)-1, inhibiting OPN and TNAP. RNA interference and chemical inhibition revealed that cdc42/Rac1 non-canonical signalling activation of USF1 supports OPN expression via association with OPN chromatin. A normal function of LRP6 was shown to be necessary to restrain vascular smooth muscle lineage non-canonical signals that promote osteochondrogenic differentiation, by inhibiting USF1 and upregulating Jmjd6, a demethylase [80]. Consequently, the identification of key factors involved in SMC characteristics may provide new insight for reducing plaque rupture and vascular calcification. Further studies need to be done to thoroughly understand the precise role of β-catenin signalling in SMC behaviour.

## 7. Wnt participation in macrophage activation

A dysfunctional endothelium and inflamed vessel wall ultimately lead to the recruitment of circulating monocytes, rolling, adhesion and migration to the subendothelial layer where they ultimately differentiate into tissue macrophages. A higher level of Wnt5a was found in macrophage-rich areas [59]. Wnt5a and its receptor Fzd5 were expressed in different subtypes of macrophages, suggesting an autocrinal effect between Wnt5a and macrophages [81]. ox-LDL was identified to increase Wnt5a expression in human monocyte-derived macrophages [82]. Toll-like receptor 4 mediated Wnt5a expression in macrophages and subsequent immune response. Through secretion of multiple pro-inflammatory cytokines and chemokines like TNF*α*, IL-18 and IL-12, plaque macrophages promoted further recruitment of inflammatory cells and amplified the inflammatory response [83]. *In vitro*, Wnt5a antagonist SFRP5 could inhibit JNK signalling induced IL-6 secretion. Interestingly, Wnt5a blocked lipopolysaccharide-induced COX2 expression in a dose-dependent manner [84]. Those data indicate a dual pro- and anti-inflammatory role of Wnt5a regarding cellular context.

Ramsey *et al*. [85] performed a system biology investigation of the transcriptional regulators of the arterial plaque macrophage and their response to lipid lowering *in vivo* in both the Reversa mouse and an aortic transplant-based mouse model. They found the canonical Wnt signalling pathway may be activated in plaque macrophages during plaque regression. A higher level of LRP5 in macrophages was found in advanced plaques compared with early lesions. Borrell-Pagès *et al*. have shown that a high-cholesterol diet induces pro-inflammatory gene expression in LRP5^−/−^ mice, suggesting the inhibitory role of the Wnt/LRP5 pathway in inflammation. LRP5^−/−^ mice developed larger aortic atherosclerotic lesions than WT mice. In their study, they showed that LDL aggregates trigger LRP5 mRNA and protein expression and β-catenin dependent signalling activation in the peripheral leucocytes [87,88]. Pro-inflammatory genes including interferon *γ*, IL15, IL18 and tumour necrosis factor ligand superfamily 13b were upregulated in LRP5^−/−^ mice compared with HC WT mice, suggesting an inhibitory role of the Wnt pathway in inflammation. This evidence for an antiatherosclerotic role of Wnt signalling has outweighed the evidence for its proatherosclerotic effect.

## 8. Conclusion

The important role of Wnt signalling pathway in cardiovascular physiology and disease has been demonstrated by numerous studies. In this review, we focused on the role of Wnt cascade in multiple pathophysiological mechanisms that take part during atherosclerosis progression, including lipid deposition, cell proliferation and cell migration. Although the results of these studies at times contradict each other, by far the majority agree on an anti-atherosclerotic role of Wnt signalling. However, Wnt signalling's intricate interaction with other molecular pathways and the formation of a complex network of signalling pathways has barred efforts in targeting specific Wnt pathway components for therapeutic intervention. The evidence suggests that the loss of Wnt signalling is the predominant defect in atherosclerosis, and hence improving Wnt activity may help with reducing plaque burden. However, there appears to be a narrow window for the therapeutic use of Wnt activators, as their excessive use has been shown to be deleterious. Thus, caution should be given in the use of Wnt activators in vascular disease and their application should be carefully evaluated on an individual basis, an approach known as precision medicine, which has shown growing popularity.

## Data accessibility

Data referenced in the paper are available in the previously published papers in the list below.

## References

[1] Cohen ED, Tian Y, Morrisey EE 2008 Wnt signaling: an essential regulator of cardiovascular differentiation, morphogenesis and progenitor self-renewal. Development 135, 789–798. (10.1242/dev.016865) 18263841

[2] de Jaime-Soguero A, Abreu de Oliveira WA, Lluis F 2018 The pleiotropic effects of the canonical wnt pathway in early development and pluripotency. Genes 9, 93 (10.3390/genes9020093) PMC5852589 29443926

[3] Lecarpentier Y, Schussler O, Hébert JL, Vallée A 2019 Multiple targets of the canonical WNT/β-Catenin signaling in cancers. Front. Oncol. 9, 1248 (10.3389/fonc.2019.01248) 31803621 PMC6876670

[4] Foulquier S, Daskalopoulos EP, Lluri G, Hermans KCM, Deb A, Blankesteijn WM 2018 WNT signaling in cardiac and vascular disease. Pharmacol. Rev. 70, 68–141. (10.1124/pr.117.013896) 29247129 PMC6040091

[5] Brade T, Männer J, Kühl M 2006 The role of Wnt signalling in cardiac development and tissue remodelling in the mature heart. Cardiovascular Research 72, 198–209. (10.1016/j.cardiores.2006.06.025) 16860783

[6] Bentzon JF, Otsuka F, Virmani R, Falk E 2014 Mechanisms of plaque formation and rupture. Circ. Res. 114, 1852–1866. (10.1161/CIRCRESAHA.114.302721) 24902970

[7] Arbustini E, Dal Bello B, Morbini P, Burke AP, Bocciarelli M, Specchia G, Virmani R 1999 Plaque erosion is a major substrate for coronary thrombosis in acute myocardial infarction. Heart (British Cardiac Society) 82, 269–272. 10455073 10.1136/hrt.82.3.269 PMC1729173

[8] Mittal B, Mishra A, Srivastava A, Kumar S, Garg N 2014 Matrix metalloproteinases in coronary artery disease. Adv. Clin. Chem. 64, 1–72. (10.1016/B978-0-12-800263-6.00001-X) 24938016

[9] Silvestre-Roig C et al. 2019 Externalized histone H4 orchestrates chronic inflammation by inducing lytic cell death. Nature 569, 236–240. (10.1038/s41586-019-1167-6) 31043745 PMC6716525

[10] White SJ, Newby AC, Johnson TW 2016 Endothelial erosion of plaques as a substrate for coronary thrombosis. Thromb. Haemost. 115, 509–519. (10.1160/th15-09-0765) 26791872

[11] Tamai K, Semenov M, Kato Y, Spokony R, Liu C, Katsuyama Y, Hess F, Saint-Jeannet J-P, He X 2000 LDL-receptor-related proteins in Wnt signal transduction. Nature 407, 530–535. (10.1038/35035117) 11029007

[12] Mani A et al. 2007 LRP6 mutation in a family with early coronary disease and metabolic risk factors. Science 315, 1278–1282. (10.1126/science.1136370) 17332414 PMC2945222

[13] Deak II 1978 Thoracic duplications in the mutant wingless of Drosophila and their effect on muscles and nerves. Dev. Biol. 66, 422–441. (10.1016/0012-1606(78)90249-X) 100357

[14] Nusse R, Varmus HE 1982 Many tumors induced by the mouse mammary tumor virus contain a provirus integrated in the same region of the host genome. Cell 31, 99–109. (10.1016/0092-8674(82)90409-3) 6297757

[15] Sato T et al. 2011 Paneth cells constitute the niche for Lgr5 stem cells in intestinal crypts. Nature 469, 415–418. (10.1038/nature09637) 21113151 PMC3547360

[16] Ng LF, Kaur P, Bunnag N, Suresh J, Sung ICH, Tan QH, Gruber J, Tolwinski NS 2019 WNT signaling in disease. Cells 8, 826. 10.3390/cells8080826 PMC6721652 31382613

[17] Many AM, Brown AM 2014 Both canonical and non-canonical Wnt signaling independently promote stem cell growth in mammospheres. PLoS ONE 9, e101800 (10.1371/journal.pone.0101800) 25019931 PMC4096729

[18] Mikels AJ, Nusse R 2006 Purified Wnt5a protein activates or inhibits beta-catenin-TCF signaling depending on receptor context. PLoS Biol. 4, e115 (10.1371/journal.pbio.0040115) 16602827 PMC1420652

[19] Tao Q et al. 2005 Maternal wnt11 activates the canonical wnt signaling pathway required for axis formation in Xenopus embryos. Cell 120, 857–871. (10.1016/j.cell.2005.01.013) 15797385

[20] Logan CY, Nusse R 2004 The Wnt signaling pathway in development and disease. Annu. Rev. Cell Dev. Biol. 20, 781–810. (10.1146/annurev.cellbio.20.010403.113126) 15473860

[21] Kohn AD, Moon RT 2005 Wnt and calcium signaling: beta-catenin-independent pathways. Cell Calcium. 38, 439–446. (10.1016/j.ceca.2005.06.022) 16099039

[22] De A 2011 Wnt/Ca^2+^ signaling pathway: a brief overview. Acta Biochimica et Biophysica Sinica. 43, 745–756. (10.1093/abbs/gmr079) 21903638

[23] Petrova IM, Malessy MJ, Verhaagen J, Fradkin LG, Noordermeer JN 2014 Wnt signaling through the Ror receptor in the nervous system. Mol. Neurobiol. 49, 303–315. (10.1007/s12035-013-8520-9) 23990374

[24] Bijakowski C et al. 2012 Sizzled is unique among secreted frizzled-related proteins for its ability to specifically inhibit bone morphogenetic protein-1 (BMP-1)/tolloid-like proteinases. J. Biol. Chem. 287, 33 581–33 593. (10.1074/jbc.M112.380816) PMC3463369 22825851

[25] Niida A, Hiroko T, Kasai M, Furukawa Y, Nakamura Y, Suzuki Y, Sugano S, Akiyama T 2004 DKK1, a negative regulator of Wnt signaling, is a target of the beta-catenin/TCF pathway. Oncogene 23, 8520–8526. (10.1038/sj.onc.1207892) 15378020

[26] Lee AY, He B, You L, Xu Z, Mazieres J, Reguart N, Mikami I, Batra S, Jablons DM 2004 Dickkopf-1 antagonizes Wnt signaling independent of beta-catenin in human mesothelioma. Biochem. Biophys. Res. Commun. 323, 1246–1250. (10.1016/j.bbrc.2004.09.001) 15451431

[27] Mikheev AM, Mikheeva SA, Liu B, Cohen P, Zarbl H 2004 A functional genomics approach for the identification of putative tumor suppressor genes: Dickkopf-1 as suppressor of HeLa cell transformation. Carcinogenesis 25, 47–59. (10.1093/carcin/bgg190) 14555616

[28] Cruciat CM, Niehrs C 2013 Secreted and transmembrane wnt inhibitors and activators. Cold Spring Harb. Perspect. Biol. 5, a015081 (10.1101/cshperspect.a015081) 23085770 PMC3578365

[29] Wu W, Glinka A, Delius H, Niehrs C 2000 Mutual antagonism between *dickkopf1* and *dickkopf2* regulates Wnt/beta-catenin signalling. Curr. Biol. 10, 1611–1614. (10.1016/S0960-9822(00)00868-X) 11137016

[30] Mao B, Niehrs C 2003 Kremen2 modulates Dickkopf2 activity during Wnt/LRP6 signaling. Gene 302, 179–183. (10.1016/S0378-1119(02)01106-X) 12527209

[31] Yu B et al. 2017 A cytokine-like protein Dickkopf-related protein 3 is atheroprotective. Circulation 136, 1022–1036. (10.1161/CIRCULATIONAHA.117.027690) 28674110 PMC5598907

[32] Karamariti E et al. 2018 DKK3 (Dickkopf 3) alters atherosclerotic plaque phenotype involving vascular progenitor and fibroblast differentiation into smooth muscle cells. Arterioscler Thromb. Vasc. Biol. 38, 425–437. (10.1161/ATVBAHA.117.310079) 29284609

[33] Cheng WL et al. 2017 Dickkopf-3 ablation attenuates the development of atherosclerosis in ApoE-Deficient Mice. J. Am. Heart Assoc. 6, e004690 (10.1161/jaha.116.004690) 28219919 PMC5523766

[34] Patel S et al. 2018 Structural and functional analysis of Dickkopf 4 (Dkk4): new insights into Dkk evolution and regulation of Wnt signaling by Dkk and Kremen proteins. J. Biol. Chem. 293, 12 149–12 166. (10.1074/jbc.RA118.002918) PMC6078440 29925589

[35] Peng S, Zhu Y, Lü B, Xu F, Li X, Lai M 2013 TCF7L2 gene polymorphisms and type 2 diabetes risk: a comprehensive and updated meta-analysis involving 121,174 subjects. Mutagenesis 28, 25–37. (10.1093/mutage/ges048) 23188737

[36] Uma Jyothi K, Jayaraj M, Subburaj KS, Prasad KJ, Kumuda I, Lakshmi V, Reddy BM 2013 Association of TCF7L2 gene polymorphisms with T2DM in the population of Hyderabad, India. PLoS ONE 8, e60212 (10.1371/journal.pone.0060212) 23577093 PMC3618330

[37] Bielinski SJ, Pankow JS, Folsom AR, North KE, Boerwinkle E 2008 TCF7L2 single nucleotide polymorphisms, cardiovascular disease and all-cause mortality: the Atherosclerosis Risk in Communities (ARIC) study. Diabetologia 51, 968–970. (10.1007/s00125-008-1004-1) 18437354 PMC2597203

[38] Sousa AG, Marquezine GF, Lemos PA, Martinez E, Lopes N, Hueb WA, Krieger JE, Pereira AC 2009 TCF7L2 polymorphism rs7903146 is associated with coronary artery disease severity and mortality. PLoS ONE 4, e7697 (10.1371/journal.pone.0007697) 19924244 PMC2773425

[39] Kucharska-Newton AM et al. 2010 Role of BMI in the association of the TCF7L2 rs7903146 variant with coronary heart disease: the Atherosclerosis Risk in Communities (ARIC) Study. J Obes. 2010, 1–5. (10.1155/2010/651903) PMC2925094 20798759

[40] Corella D et al. 2013 Mediterranean diet reduces the adverse effect of the TCF7L2-rs7903146 polymorphism on cardiovascular risk factors and stroke incidence: a randomized controlled trial in a high-cardiovascular-risk population. Diab. Care. 36, 3803–3811. (10.2337/dc13-0955) PMC3816851 23942586

[41] Muendlein A, Saely CH, Geller-Rhomberg S, Sonderegger G, Rein P, Winder T, Beer S, Vonbank A, Drexel H,. 2011 Single nucleotide polymorphisms of TCF7L2 are linked to diabetic coronary atherosclerosis. PLoS ONE 6, e17978 (10.1371/journal.pone.0017978) 21423583 PMC3058059

[42] Gaudio A, Privitera F, Pulvirenti I, Canzonieri E, Rapisarda R, Fiore CE 2014 The relationship between inhibitors of the Wnt signalling pathway (sclerostin and Dickkopf-1) and carotid intima-media thickness in postmenopausal women with type 2 diabetes mellitus. Diab. Vasc. Dis. Res. 11, 48–52. (10.1177/1479164113510923) 24227537

[43] Seifert-Held T, Pekar T, Gattringer T, Simmet NE, Scharnagl H, Stojakovic T, Fazekas F, Storch MK 2011 Circulating Dickkopf-1 in acute ischaemic stroke and clinically stable cerebrovascular disease. Atherosclerosis 218, 233–237. (10.1016/j.atherosclerosis.2011.05.015) 21663914

[44] Ress C et al. 2018 Circulating Wnt inhibitory factor 1 levels are associated with development of cardiovascular disease. Atherosclerosis 273, 1–7. (10.1016/j.atherosclerosis.2018.03.045) 29649633

[45] Ueland T et al. 2019 Admission levels of DKK1 (Dickkopf-1) are associated with future cardiovascular death in patients with acute coronary syndromes. Arterioscler. Thromb. Vasc. Biol. 39, 294–302. (10.1161/ATVBAHA.118.311042) 30580572

[46] Zhu Z et al. 2019 Serum Dkk-1 (Dickkopf-1) is a potential biomarker in the prediction of clinical outcomes among patients with acute ischaemic stroke. Arterioscler. Thromb. Vasc. Biol. 39, 285–293. (10.1161/ATVBAHA.118.311960) 30580563

[47] Boucher P, Matz RL, Terrand J 2020 Atherosclerosis: gone with the Wnt? Atherosclerosis 301, 15–22. (10.1016/j.atherosclerosis.2020.03.024) 32289618

[48] Tomaszewski M et al. 2009 A common variant in low-density lipoprotein receptor-related protein 6 gene (LRP6) is associated with LDL-cholesterol. Arterioscler. Thromb. Vasc. Biol. 29, 1316–1321. (10.1161/ATVBAHA.109.185355) 19667113 PMC2814817

[49] Singh R, Smith E, Fathzadeh M, Liu W, Go GW, Subrahmanyan L, Faramarzi S, Mckenna W, Mani A 2013 Rare nonconservative LRP6 mutations are associated with metabolic syndrome. Hum. Mut. 34, 1221–1225. (10.1002/humu.22360) 23703864 PMC3745535

[50] Guo J et al. 2016 Mutant LRP6 impairs endothelial cell functions associated with familial normolipidemic coronary artery disease. Int. J. Mol. Sci. 17, 1173 (10.3390/ijms17071173) PMC4964544 27455246

[51] Xu S et al. 2014 The LRP6 rs2302685 polymorphism is associated with increased risk of myocardial infarction. Lipids Health Dis. 13, 94 (10.1186/1476-511X-13-94) 24906453 PMC4059096

[52] Sarzani R, Salvi F, Bordicchia M, Guerra F, Battistoni I, Pagliariccio G, Carbonari L, Dessì-Fulgheri P, Rappelli A 2011 Carotid artery atherosclerosis in hypertensive patients with a functional LDL receptor-related protein 6 gene variant. Nutr. Metab. Cardiovasc. Dis. 21, 150–156. (10.1016/j.numecd.2009.08.004) 19833493

[53] Liu W, Mani S, Davis NR, Sarrafzadegan N, Kavathas PB, Mani A 2008 Mutation in EGFP domain of LDL receptor-related protein 6 impairs cellular LDL clearance. Circ. Res. 103, 1280–1288. (10.1161/CIRCRESAHA.108.183863) 18948618 PMC3426315

[54] Ye ZJ, Go GW, Singh R, Liu W, Keramati AR, Mani A 2012 LRP6 protein regulates low density lipoprotein (LDL) receptor-mediated LDL uptake. J. Biol. Chem. 287, 1335–1344. (10.1074/jbc.M111.295287) 22128165 PMC3256876

[55] Go GW, Srivastava R, Hernandez-Ono A, Gang G, Smith SB, Booth CJ, Ginsberg HN, Mani A 2014 The combined hyperlipidemia caused by impaired Wnt-LRP6 signaling is reversed by Wnt3a rescue. Cell Metabolism 19, 209–220. (10.1016/j.cmet.2013.11.023) 24506864 PMC3920193

[56] Kirsch N, Chang LS, Koch S, Glinka A, Dolde C, Colozza G, Benitez MDJ, De Robertis EM, Niehrs C 2017 Angiopoietin-like 4 Is a Wnt signaling antagonist that promotes LRP6 turnover. Dev. Cell 43, 71–82.e6. (10.1016/j.devcel.2017.09.011) 29017031

[57] Bretón-Romero R et al. 2016 Endothelial dysfunction in human diabetes is mediated by Wnt5a-JNK signaling. Arterioscler. Thromb. Vasc. Biol. 36, 561–569. (10.1161/ATVBAHA.115.306578) 26800561 PMC4913891

[58] Skaria T, Burgener J, Bachli E, Schoedon G 2016 IL-4 causes hyperpermeability of vascular endothelial cells through Wnt5A signaling. PLoS ONE 11, e0156002 (10.1371/journal.pone.0156002) 27214384 PMC4877093

[59] Christman MA II, Goetz DJ, Dickerson E, McCall KD, Lewis CJ, Benencia F, Silver MJ, Kohn LD, Malgor R 2008 Wnt5a is expressed in murine and human atherosclerotic lesions. Am. J. Physiol. Heart Circ. Physiol. 294, H2864–H2870. (10.1152/ajpheart.00982.2007) 18456733

[60] Kim J, Kim J, Kim DW, Ha Y, Ihm MH, Kim H, Song K, Lee I 2010 Wnt5a induces endothelial inflammation via beta-catenin-independent signaling. J. Immunol. 185, 1274–1282. (10.4049/jimmunol.1000181) 20554957

[61] Relling I et al. 2018 Role of wnt5a in metabolic inflammation in humans. J. Clin. Endocrinol. Metabolism 103, 4253–4264. (10.1210/jc.2018-01007) 30137542

[62] Akoumianakis I et al. 2019 Adipose tissue-derived WNT5A regulates vascular redox signaling in obesity via USP17/RAC1-mediated activation of NADPH oxidases. Sci. Transl. Med. 11, eaav5055 (10.1126/scitranslmed.aav5055) 31534019 PMC7212031

[63] Cheng CW, Yeh JC, Fan TP, Smith SK, Charnock-Jones DS 2008 Wnt5a-mediated non-canonical Wnt signalling regulates human endothelial cell proliferation and migration. Biochem. Biophys. Res. Commun. 365, 285–290. (10.1016/j.bbrc.2007.10.166) 17986384

[64] Ueland T et al. 2009 Dickkopf-1 enhances inflammatory interaction between platelets and endothelial cells and shows increased expression in atherosclerosis. Arterioscler. Thromb. Vasc. Biol. 29, 1228–1234. (10.1161/ATVBAHA.109.189761) 19498175

[65] Di M et al. 2017 Dickkopf1 destabilizes atherosclerotic plaques and promotes plaque formation by inducing apoptosis of endothelial cells through activation of ER stress. Cell Death & Disease 8, e2917 (10.1038/cddis.2017.277) 28703797 PMC5550842

[66] Souilhol C, Harmsen MC, Evans PC, Krenning G 2018 Endothelial–mesenchymal transition in atherosclerosis. Cardiovasc. Res. 114, 565–577. (10.1093/cvr/cvx253) 29309526

[67] Cheng SL, Shao JS, Behrmann A, Krchma K, Towler DA 2013 Dkk1 and MSX2-Wnt7b signaling reciprocally regulate the endothelial-mesenchymal transition in aortic endothelial cells. Arterioscler. Thromb. Vasc. Biol. 33, 1679–1689. (10.1161/ATVBAHA.113.300647) 23685555 PMC3837473

[68] Busceti CL et al. 2017 Dickkopf-3 Upregulates VEGF in cultured human endothelial cells by activating activin receptor-like kinase 1 (ALK1) pathway. Front. Pharmacol. 8, 111 (10.3389/fphar.2017.00111) 28352232 PMC5348502

[69] Visel A et al. 2010 Targeted deletion of the 9p21 non-coding coronary artery disease risk interval in mice. Nature 464, 409–412. (10.1038/nature08801) 20173736 PMC2938076

[70] Sano H, Sudo T, Yokode M, Murayama T, Kataoka H, Takakura N, Nishikawa S, Nishikawa S-I, Kita T 2001 Functional blockade of platelet-derived growth factor receptor-beta but not of receptor-alpha prevents vascular smooth muscle cell accumulation in fibrous cap lesions in apolipoprotein E-deficient mice. Circulation 103, 2955–2960. (10.1161/01.CIR.103.24.2955) 11413086

[71] Quasnichka H, Slater SC, Beeching CA, Boehm M, Sala-Newby GB, George SJ 2006 Regulation of smooth muscle cell proliferation by beta-catenin/T-cell factor signaling involves modulation of cyclin D1 and p21 expression. Circ. Res. 99, 1329–1337. (10.1161/01.RES.0000253533.65446.33) 17122440

[72] Uglow EB, Slater S, Sala-Newby GB, Aguilera-Garcia CM, Angelini GD, Newby AC, George SJ 2003 Dismantling of cadherin-mediated cell-cell contacts modulates smooth muscle cell proliferation. Circ. Res. 92, 1314–1321. (10.1161/01.RES.0000079027.44309.53) 12775583

[73] Srivastava R, Zhang J, Go GW, Narayanan A, Nottoli TP, Mani A 2015 Impaired LRP6-TCF7L2 activity enhances smooth muscle cell plasticity and causes coronary artery disease. Cell Rep. 13, 746–759. (10.1016/j.celrep.2015.09.028) 26489464 PMC4626307

[74] Srivastava R et al. 2019 TCF7L2 (transcription factor 7-like 2) regulation of GATA6 (GATA-binding protein 6)-dependent and -independent vascular smooth muscle cell plasticity and intimal hyperplasia. Arterioscler. Thromb. Vasc. Biol. 39, 250–262. (10.1161/ATVBAHA.118.311830) 30567484 PMC6365015

[75] Williams H, Mill CA, Monk BA, Hulin-Curtis S, Johnson JL, George SJ 2016 Wnt2 and WISP-1/CCN4 induce intimal thickening via promotion of smooth muscle cell migration. Arterioscler. Thromb. Vasc. Biol. 36, 1417–1424. (10.1161/ATVBAHA.116.307626) 27199447

[76] Chow W, Hou G, Bendeck MP 2008 Glycogen synthase kinase 3beta regulation of nuclear factor of activated T-cells isoform c1 in the vascular smooth muscle cell response to injury. Exp. Cell Res. 314, 2919–2929. (10.1016/j.yexcr.2008.07.010) 18675800

[77] Mill C, George SJ 2012 Wnt signalling in smooth muscle cells and its role in cardiovascular disorders. Cardiovasc. Res. 95, 233–240. (10.1093/cvr/cvs141) 22492675

[78] Wang X, Xiao Y, Mou Y, Zhao Y, Blankesteijn WM, Hall JL 2002 A role for the beta-catenin/T-cell factor signaling cascade in vascular remodeling. Circ. Res. 90, 340–347. (10.1161/hh0302.104466) 11861424

[79] Shao JS, Cheng SL, Pingsterhaus JM, Charlton-Kachigian N, Loewy AP, Towler DA 2005 Msx2 promotes cardiovascular calcification by activating paracrine Wnt signals. J. Clin. Invest. 115, 1210–1220. (10.1172/JCI24140) 15841209 PMC1077175

[80] Cheng SL et al. 2015 Vascular smooth muscle LRP6 limits arteriosclerotic calcification in diabetic LDLR-/- mice by restraining noncanonical Wnt signals. Circ. Res. 117, 142–156. (10.1161/CIRCRESAHA.117.306712) 26034040 PMC4490945

[81] Blumenthal A, Ehlers S, Lauber J, Buer J, Lange C, Goldmann T, Heine H, Brandt E, Reiling N 2006 The Wingless homolog WNT5A and its receptor Frizzled-5 regulate inflammatory responses of human mononuclear cells induced by microbial stimulation. Blood 108, 965–973. (10.1182/blood-2005-12-5046) 16601243

[82] Bhatt PM, Malgor R 2014 Wnt5a: a player in the pathogenesis of atherosclerosis and other inflammatory disorders. Atherosclerosis 237, 155–162. (10.1016/j.atherosclerosis.2014.08.027) 25240110 PMC4252768

[83] Ohta H, Wada H, Niwa T, Kirii H, Iwamoto N, Fujii H, Saito K, Sekikawa K, Seishima M 2005 Disruption of tumor necrosis factor-alpha gene diminishes the development of atherosclerosis in ApoE-deficient mice. Atherosclerosis 180, 11–17. (10.1016/j.atherosclerosis.2004.11.016) 15823270

[84] Halleskog C, Schulte G 2013 WNT-3A and WNT-5A counteract lipopolysaccharide-induced pro-inflammatory changes in mouse primary microglia. J. Neurochem. 125, 803–808. (10.1111/jnc.12250) 23534675

[85] Ramsey SA, Vengrenyuk Y, Menon P, Podolsky I, Feig JE, Aderem A, Fisher EA, Gold ES 2014 Epigenome-guided analysis of the transcriptome of plaque macrophages during atherosclerosis regression reveals activation of the Wnt signaling pathway. PLoS Genetics 10, e1004828 (10.1371/journal.pgen.1004828) 25474352 PMC4256277

[86] Busceti CL et al. 2017 Dickkopf-3 upregulates VEGF in cultured human endothelial cells by activating activin receptor-like kinase 1 (ALK1) pathway. Front. Pharmacol. 8, 111. 28352232 10.3389/fphar.2017.00111 PMC5348502

[87] Borrell-Pagès M, Carolina Romero J, Badimon L 2015 LRP5 and plasma cholesterol levels modulate the canonical Wnt pathway in peripheral blood leukocytes. Immunol. Cell Biol. 93, 653–661. (10.1038/icb.2015.41) 25748163

[88] Borrell-Pagès M, Romero JC, Badimon L 2015 LRP5 deficiency down-regulates Wnt signalling and promotes aortic lipid infiltration in hypercholesterolaemic mice. J. Cell. Mol. Med. 19, 770–777. (10.1111/jcmm.12396) 25656427 PMC4395191

